# Red Blood Cells and Human Aging: Exploring Their Biomarker Potential

**DOI:** 10.3390/diagnostics15161993

**Published:** 2025-08-08

**Authors:** Roula P. Kyriacou, Sapha Shibeeb

**Affiliations:** School of Health and Biomedical Sciences, RMIT University, P.O. Box 71, Bundoora, Melbourne, VIC 3083, Australia; roula.kyriacou@rmit.edu.au

**Keywords:** red blood cells, aging, biomarker

## Abstract

Aging is a complex biological process marked by progressive physiological decline with increasing vulnerability to diseases such as cardiovascular disorders, neurodegenerative conditions, and metabolic syndromes. Identifying reliable biomarkers of aging is essential for assessing biological age, predicting health outcomes, and guiding interventions to promote healthy aging. Among various candidate biomarkers, red blood cells (RBCs) offer a unique and accessible window into the aging process due to their abundance, finite lifespan, and responsiveness to systemic changes. This review examines the potential of RBCs as biomarkers of aging by exploring their age-associated morphological, functional, and biochemical alterations. Age-related reduction in key haematological parameters such as RBC count, haemoglobin concentration, and haematocrit, and increases in mean cell volume (MCV) and red cell distribution width (RDW), reflect underlying shifts in erythropoiesis and cellular turnover. Functional changes include reduced oxygen-carrying capacity, decreased deformability, diminished ATP release, and increased RBC aggregation, all of which may impair both macrocirculatory and microcirculatory flow and tissue oxygenation. Biochemically, aging RBCs exhibit altered membrane lipid and protein composition, reduced membrane fluidity, and diminished antioxidant and enzymatic activity, contributing to cellular senescence and clearance. Despite these promising indicators, challenges persist in establishing RBC parameters as definitive biomarkers of aging. Inter-individual and intra-individual variability and storage-related artifacts complicate their use. In conclusion, RBCs present a compelling, though currently underutilized, avenue for aging biomarker research. Further longitudinal validation and mechanistic research are essential to support the clinical utility of RBC parameters as biomarkers of aging.

## 1. Introduction

Human aging is a multifaceted process characterized by a gradual decline in physiological function, leading to an increased susceptibility of age related conditions and mortality [1]. This deterioration is a primary risk factor for diabetes, cardiovascular disorders, and neurodegenerative diseases [1]. Understanding the mechanisms underlying aging and identifying reliable biomarkers to track its progression are essential for predicting health outcomes and developing effective strategies to promote healthy aging [2]. Developing reliable aging biomarkers can enable a more precise prediction of an individual’s aging condition and aid in the development of anti-aging strategies [2]. Among the various potential biomarkers, RBCs have attracted attention due to their abundance, deformability, and finite lifespan (approx. 120 days) in humans [3]. This limited lifespan suggests that RBCs may be sensitive indicators of age-related changes occurring at both cellular and systemic levels. This comprehensive narrative review aims to explore the current understanding of RBCs as biomarkers of human aging.

## 2. Defining Biomarkers of Aging

Biomarkers of aging are biological indicators that reflect the structural or functional decline in reductions in cellular regeneration, metabolic efficiency, and overall decline in organ function, associated with advancing age [2]. Ideally, these biomarkers should possess several key characteristics to be useful in gerontology research. They should be capable of predicting an individual’s functional capacity at a later age more effectively than chronological age alone, essentially providing a measure of an individual’s true biological age which may differ from their age in years [4].

The distinction between chronological age and biological age is fundamental to the study of aging. Chronological age refers to the amount of time an individual has lived since birth, while biological age reflects their physiological state relative to others of the same chronological age [5]. Individuals of the same chronological age can show significant differences in their susceptibility to age-related diseases and mortality, highlighting the fact that chronological age is an imperfect surrogate for the underlying biological aging processes.

A variety of biomarkers have been investigated as indicators of aging, including molecular markers such as telomere length and DNA methylation patterns, clinical markers like inflammatory proteins (e.g., CRP, IL-6), and functional measures such as grip strength and walking speed ki [6,7]. Laboratory-based biomarkers encompass molecular assessments, such as telomere length, which reflects cellular aging, and epigenetic clocks, which estimate biological age based on DNA methylation patterns. Routine clinical biomarkers, commonly measured using standardized methods in accredited laboratories, include complete blood counts and inflammatory markers. These also serve as surrogate indicators of organ function, providing insights into the health status of various systems. High-throughput molecular analyses—particularly proteomics and metabolomics—have facilitated the identification of age-associated alterations in mature red blood cells, including changes in membrane proteins, metabolic intermediates, and oxidative stress markers, which may serve as potential biomarkers of aging [8,9]. In contrast, genomic and transcriptomic approaches are more applicable at the level of erythroid progenitor cells, offering upstream insights into aging-related changes in erythropoiesis and red cell production [10]. Although promising, omics-based biomarkers currently remain confined to research settings due to limited validation and technical challenges. Non-molecular phenotypic biomarkers describe observable physiological functions of the body, such as physical capability (e.g., grip strength, gait speed) and organ function (e.g., measures of respiratory or cognitive function) [11].

## 3. Aging and Red Cell Production and Regulation

The production of RBCs, or erythropoiesis, is a tightly regulated physiological process that ensures adequate oxygen transport and tissue perfusion throughout the body. As an individual ages, alterations in erythropoiesis can contribute to observed changes in red cell morphology and function, underscoring the importance of understanding the regulatory mechanisms that govern red cell production in the context of aging (Table 1).

RBC production begins with haematopoietic stem cells (HSCs) in the bone marrow, which differentiate through a series of progenitor stages to form mature erythrocytes. This process is regulated by several intrinsic and extrinsic factors, with erythropoietin (EPO) being the principal hormonal driver of red cell lineage commitment and proliferation. EPO is produced primarily in the kidneys in response to hypoxia and acts on erythroid progenitors by binding to the EPO receptor, promoting survival, proliferation, and differentiation [12].

Aging is associated with a decline in the regenerative capacity of HSCs, often described as “stem cell exhaustion” [13]. Aged HSCs demonstrate reduced self-renewal and diminished responsiveness to growth factors such as EPO [14]. These changes may contribute to the observed decline in red blood cell count, haemoglobin concentration, and haematocrit in older adults. Furthermore, aged bone marrow exhibits increased adiposity and a pro-inflammatory microenvironment, both of which negatively affect erythropoietic proliferation and differentiation [15].

With advancing age, there is a well-documented increase in circulating inflammatory cytokines, contributing to a state of chronic low-grade inflammation commonly referred to as inflammaging [16]. Inflammatory cytokines such as interleukin-1, tumour necrosis factor-alpha, and interferon-gamma can inhibit erythroid progenitor proliferation and suppress EPO production, contributing to anemia of chronic disease, a condition that becomes more prevalent with age [17]. These inflammatory mediators activate intracellular signaling pathways such as NF-κB, JAK/STAT that interfere with erythroid transcription factors such as GATA-1 and erythroid Krüppel-like factor (EKLF), thereby disrupting normal erythropoiesis [18,19].

Iron availability also plays a critical role in red cell production. With aging, functional iron deficiency may develop due to impaired absorption, chronic inflammation (via hepcidin-mediated sequestration), or comorbid conditions such as gastrointestinal disorders [20]. A key mediator in this process is hepcidin, a hepatic peptide hormone and acute-phase reactant that regulates systemic iron homeostasis. Hepcidin expression is upregulated by pro-inflammatory cytokines, particularly interleukin-6, in chronic low-grade inflammation commonly observed in older adults [21,22]. Once elevated, hepcidin acts by degrading ferroportin, the sole known cellular iron exporter located on enterocytes and macrophages, thereby reducing dietary iron absorption and iron release from stores [23]. This leads to decreased systemic iron availability and contributes to the development of hypoproliferative anemia, even in the presence of adequate iron stores—a hallmark of anemia of chronic disease and aging.

## 4. Age-Related Changes in Red Blood Cells

**Figure 1:**
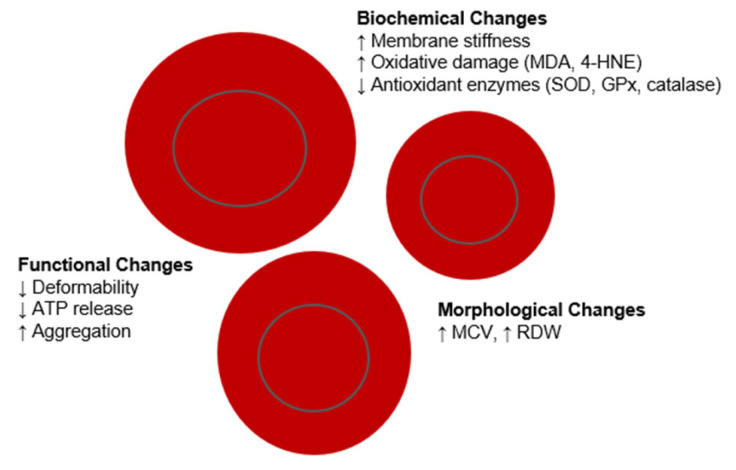
Age-related changes in red blood cells.

RBCs undergo functional, morphological and biochemical changes with aging (Figure 1). Many studies have reported correlation between various RBC parameters and chronological age in humans. Routine haematological indices such as RBC count, haemoglobin, and haematocrit have been shown to decrease with increasing age. This decline may reflect a reduced capacity of the bone marrow to produce new RBCs [24]. Conversely, MCV, and mean cell haemoglobin tend to increase with age [25,26].

The MCV reflects the average size and volume of circulating RBCs and is a key parameter used in the classification of anemia. It has also been proposed as a prognostic marker in non-anemic populations, with associations reported in conditions such as colorectal and esophageal cancer, chronic kidney disease, and cardiovascular events [27].

Several studies have investigated the relationship between MCV and aging. Lee et al. [27] reported a gradual increase in MCV with age. Similarly, Chen et al. reported age-related increases in MCV [28]. Goldberg et al. reported an average annual increase of 0.184 fL in MCV in a retrospective analysis that included 3551 subjects [29]. These findings suggest an age-associated alteration in erythropoiesis, suggesting that a shortened RBC lifespan in older adults may enhance erythropoietic activity and the release of larger, younger erythrocytes leading to increasing MCV values over time. Another significant morphological change observed with aging is an increase in RDW [30]. RDW reflects anisocytosis, or the degree of variation in the size of circulating RBCs. It is calculated by dividing the standard deviation (SD) of red blood cell volumes by the MCV). RDW can be reported as an absolute value (RDW-SD) or as a percentage (RDW-%), with the latter being more widely used in routine clinical practice [31]. An increased RDW has been linked to impaired erythropoietic output, disrupted iron homeostasis, inflammatory processes, oxidative stress, and red cell senescence [32,33].

The reasons underlying the association between red cell size variability and aging are not yet fully understood. Recent studies have provided an insight into the mechanistic and prognostic basis for RDW in the context of aging. For example, Kim et al. [34] demonstrated that increased RDW is independently associated with frailty and multiple age-related outcomes, including cognitive decline and mobility impairment. Similarly, Gialluisi et al. [35] found that RDW correlated significantly with biological age measures and was elevated in individuals with Parkinson’s disease, reinforcing its potential link to neurodegenerative processes. These findings suggest RDW may serve as a sensitive, systemic indicator of subclinical aging, capable of detecting pathophysiological shifts even in the absence of overt disease.

Interestingly, an increased RDW has been associated with several age-related conditions, including frailty, cognitive decline, and increased mortality risk, suggesting its potential as a marker of overall biological aging [36].

## 5. Functional Alterations of Red Blood Cells with Age

The primary function of RBCs is to transport oxygen from the lungs to the tissues and to carry carbon dioxide back to the lungs for removal. With aging, the efficiency of this crucial function can be affected (Table 2). Haemoglobin levels, the protein within RBCs responsible for binding and carrying oxygen, tend to decrease with age [37]. This reduction in haemoglobin may potentially lead to a lower oxygen-carrying capacity in older individuals. Furthermore, the affinity of haemoglobin for oxygen, often represented by the P50 value refers to the partial pressure of oxygen at which haemoglobin is 50% saturated, may also change with age. Some studies suggest that P50 and levels of 2,3-bisphosphoglycerate (2,3-BPG) decline with age likely due to altered RBC metabolism, potentially leading to an increased affinity of haemoglobin for oxygen [38,39]. Since 2,3-BPG normally reduces haemoglobin’s oxygen affinity by stabilizing its deoxygenated form, a reduction in 2,3-BPG levels can hinder oxygen unloading at the tissue level. This shift, along with an age-related decline in haemoglobin concentration, may contribute to tissue hypoxia and negatively affect various physiological functions in older adults [40].

Another critical functional property of RBCs is their deformability, the ability to change shape and pass through narrow capillaries in the microcirculation. This property is influenced by three major factors: (1) membrane elasticity, determined by the structure and integrity of membrane proteins and lipids; (2) internal viscosity, largely influenced by haemoglobin concentration and cytoplasmic composition; and (3) the surface area–to–volume (SA:V) ratio, which allows shape change without lysis [41,42]. Reduced deformability of RBCs can hinder blood flow to organs, potentially compromising tissue oxygenation [43]. Studies have consistently shown that RBC deformability decreases with age [44,45].

Aging-related changes that reduce RBC deformability include RBC membrane lipid remodeling and protein degradation. Specific reductions in membrane-associated enzymes such as β-d-glucuronidase and neutral sialidase [46,47], oxidative stress and reduced antioxidant enzyme activity [48], have been suggested to compromise RBC membrane flexibility and surface charge. Furthermore, decline in RBC membrane fluidity and deformability has also been associated with broader metabolic dysfunction, particularly reduced ATP production [45]. ATP is essential for maintaining ion gradients, cytoskeletal organization, and membrane flexibility [49,50]. It has been reported that decreased ATP levels impair Na^+^/K^+^-ATPase activity, leading to altered intracellular cation concentrations, disrupted cell volume regulation, and a lower surface area-to-volume ratio, ultimately reducing deformability and microcirculatory efficiency [51]. It has been reported significant age-related reductions in these ATPase activities, often correlating with decreased plasma antioxidant capacity and increased intracellular Na^+^ and Ca^2+^ accumulation, suggesting a role for oxidative stress in impairing membrane transport and flexibility [52]. In addition, blood viscosity has also been shown to be impacted by progressive aging. Studies have shown that aging leads to increased blood viscosity and reduced red cell deformability [53,54]. Furthermore, RBCs of older healthy individuals exhibit increased membrane stiffness and greater adhesive properties compared to those from younger individuals. These changes are associated with age-related alterations in membrane composition leading to impaired deformability and microcirculatory flow [3,42,55,56].

Another functional property of RBCs that changes with age is their tendency to aggregate, forming clumps that can affect blood flow. Studies have reported RBC aggregation generally increases with age [53]. Older and denser RBCs have been shown to exhibit higher aggregation forces [57]. This increased aggregation in older individuals can lead to elevated blood viscosity, potentially hindering blood flow, particularly in the microcirculation, and increasing the risk of vascular events. Changes in the levels of membrane sialic acid [58], which contributes to the RBC surface charge, and alterations in the size of the depletion layer surrounding RBCs are thought to contribute to this increased aggregation tendency in older cells. Changes in the deformability of RBCs and the presence of protein fibril aggregates on their surface have been implicated in neurodegenerative diseases, such as Alzheimer’s disease [59,60].

## 6. Biochemical Modifications in Red Blood Cells During Organismal Aging

The aging process in RBCs is accompanied by progressive biochemical alterations that affect membrane structure and stability, enzymatic function, and redox balance [61,62]. These changes contribute to the reduced deformability, shortened lifespan, and increased clearance of RBCs in older adults.

The lipid bilayer of the RBC membrane undergoes well-documented compositional changes with age. Studies have shown an increase in total cholesterol and phospholipid content, along with a shift in fatty acid composition, characterized by elevated levels of saturated fatty acids and a reduction in polyunsaturated fatty acids [63,64]. These changes reduce membrane fluidity, making RBCs more rigid and susceptible to mechanical stress [58]. Furthermore, lipid peroxidation, a marker of oxidative stress, has been found to increase in healthy elderly humans [65]. These age-related modifications in the lipid composition of the RBC membrane can affect its fluidity and stability, potentially contributing to the functional decline observed in older RBCs and making them more susceptible to damage.

RBC membrane proteins also exhibit significant age-related changes. Band 3, the most abundant integral membrane protein, undergoes clustering, proteolytic cleavage, and altered glycosylation patterns over time [46]. Protein 4.1b, a cytoskeletal linker protein, undergoes deamidation to form 4.1a, and the changing ratio of these isoforms serves as a biochemical marker of RBC age. In addition, age-related variation in the expression of complement regulatory proteins on the RBC surface has been observed, potentially reducing the cell’s protection against immune-mediated lysis [66]. These age-related protein-level changes facilitate the recognition and removal of senescent RBCs from circulation via the reticuloendothelial system.

Aging is associated with increased production of intracellular reactive oxygen species (ROS), generated through mechanisms such as haemoglobin auto-oxidation and NADPH oxidase activity [67]. The resulting oxidative stress contributes to RBC membrane lipid peroxidation and protein oxidation. Age-related decline in the activity of key antioxidant enzymes, including glutathione peroxidase (GPx), superoxide dismutase (SOD), catalase, and glutathione-S-transferase (GST), as well as depletion of nonenzymatic antioxidants such as reduced glutathione (GSH) and vitamin C, has been reported in RBCs of aged individuals [68,69]. Biomarkers of oxidative stress, such as elevated levels of malondialdehyde (MDA), 4-hydroxy-2-nonenal (4-HNE), protein carbonyls, and decreased GSH/GSSG ratios, have been found to correlate with increasing chronological age [62,70]. These metabolic and redox imbalances contribute to reduced membrane fluidity and deformability, increased cellular fragility of RBCs during aging.

## 7. Comparative Assessment of Red Blood Cells and Other Aging Biomarkers

Several aging biomarkers have been investigated for their potential to measure biological aging, including molecular indicators such as telomere length and DNA methylation patterns [71]. Each of these approaches provides insight into specific aspects of the aging process, but they also present limitations related to their measurement, interpretation, and applicability [72], as seen in Table 3.

Telomere length (TL), a measure of the DNA sequence repeats at the ends of chromosomes, is often reported as a classical aging biomarker. Telomeres shorten with each cell division, and critically short telomeres trigger cellular senescence or apoptosis. While telomeres generally shorten with chronological age, using TL as a standalone biomarker presents several limitations. A primary concern is the inconsistency and often equivocal nature of the evidence surrounding its reliability. Studies have frequently yielded weak or conflicting correlations between TL and overall biological age or health outcomes, undermining its utility as a definitive marker [73,74,75]. For example, two individuals of the same chronological age could have very different TLs, and those differences do not always correspond to differences in health or biological aging. TL is influenced by multiple non-aging factors, including genetics, lifestyle (e.g., diet, smoking, exercise), stress, early life conditions (prenatal factors, maternal nutrition and health), environmental exposures, and co-morbidities. This multifactorial influence complicates the attribution of TL changes solely to biological aging. An individual may have relatively long telomeres due to genetic predisposition or low oxidative stress, while another may have much shorter telomeres due to chronic stress or illness. Not only can TL vary between individuals, but also among different cell types within the same person. Some cells divide more frequently than others or they are exposed to more oxidative stress, leading to faster telomere shortening [76]. On the other hand, certain stem or progenitor cells can maintain TL better due to telomerase activity, the enzyme that replenishes telomeres [77]. The high variability in TL thereby reduces its reliability and limits its predictive value for assessing an individual’s physiological decline and biological aging.

In addition to interpretative variability, TL is methodologically challenging to measure with accuracy [78]. Most high-throughput methods report average telomere length (aTL), which may obscure the presence of a few critically short telomeres that are believed to be the primary drivers of cellular senescence. The distribution and presence of these functionally relevant short telomeres are often not captured by conventional assays, only the average length is determined. Additional challenges include intra- and inter-assay variability, differences in tissue specificity, and limited generalizability across populations and age groups [71,79,80]. Repeated qPCR assays within the same laboratory on the same DNA sample may produce slightly different TL values on different days or by different technicians, while variations between laboratories (e.g., protocols, equipment, and calibration standards) can also significantly impact the reproducibility and comparability of TL measurements. Similarly, TL norms derived from one ethnic population may not apply to others, limiting cross-population comparisons. In addition, older individuals exhibit more interindividual variability, making population-level generalizations more challenging.

A second major class of aging biomarkers involves epigenetic clocks [81]. These clocks estimate biological age based on patterns of DNA methylation at specific CpG sites across the genome [82]. Despite their growing popularity, several limitations constrain their utility and generalizability. A primary concern is tissue specificity as methylation patterns vary across tissues. For example, biological age predictions from blood may not reflect aging in other organs like the brain or skin. This limits generalizability unless a clock is explicitly trained on multi-tissue data. Epigenetic clocks correlate well with chronological age and predict age-related health outcomes, yet their utility is constrained by limited mechanistic insight. It remains unclear what biological processes underline the methylation changes at CpG sites included in the clocks, which hinders interpretation of what an “accelerated” or “decelerated” epigenetic age truly signifies [83,84]. Many of the methylation changes may reflect the downstream effects of aging or environmental exposures rather than causal mechanisms that drive the aging processes.

Epigenetic clocks also suffer from confounding due to blood cell-type composition [85,86]. DNA methylation is highly cell-type specific and shifts in immune cell populations with age or disease may alter methylation profiles independently of intrinsic cellular aging [86]. For example, acute illnesses, inflammation, smoking, or psychosocial stresses can induce changes in DNA methylation that are not necessarily related to aging. The generalizability and specificity of epigenetic clocks across diverse populations and contexts also pose a challenge. Indeed, individuals from different genetic backgrounds, ethnicities, or geographical locations may exhibit distinct methylation patterns influenced by unique genetic variations and environmental exposures [87,88].

Moreover, the technical complexity associated with comprehensive DNA methylation profiling can limit the widespread clinical implementation of epigenetic clocks. While the cost has decreased significantly, measuring DNA methylation at hundreds or thousands of CpG sites still requires specialized equipment and considerable bioinformatics expertise for data processing and analysis [89,90].

In contrast, RBC parameters such as RDW and MCV are measured using standardised, automated analysers that are routinely used in clinical practice. These haematological indices are cost-effective, reproducible, and globally accessible. Unlike TL or epigenetic clocks, which often rely on specialized molecular techniques and freshly processed samples, RBC indices are readily available from historical and ongoing clinical datasets. This facilitates both retrospective and prospective investigations in longitudinal population studies.

Importantly, RBCs capture physiological aspects of aging not reflected in molecular clocks, such as blood rheology, oxygen transport capacity, and microvascular function. Furthermore, these parameters respond dynamically to systemic stressors including inflammation, oxidative stress, and nutrient deficiencies, aligning closely with multiple hallmarks of aging and offering potential as real-time functional biomarkers.

Another key advantage is their applicability in low-resource settings. In contrast to TL or DNA methylation assays that require advanced laboratory infrastructure and complex molecular platforms, RBC indices can be obtained using basic haematology analysers, supporting broader implementation in global health and aging research.

## 8. Limitations and Challenges in Utilizing Red Blood Cells as Aging Biomarkers

Despite the promising potential of RBCs as biomarkers of aging, several limitations and challenges need to be considered. Mature RBCs lack subcellular organelles, including a nucleus, which limits their functionality and ability to carry out complex cellular functions compared to nucleated cells. This might restrict their ability to exhibit the same hallmarks of the aging process as nucleated cells [91]. Furthermore, there is significant inter-individual and intra-individual variability in RBC parameters due to a multitude of factors such as genetics, lifestyle, diet, physical activity and environmental exposures. This variability can confound the use of RBCs as universal biomarkers of aging, making it challenging to establish clear age-related reference ranges [92,93].

Additionally, the conditions under which blood samples are stored can significantly impact RBC morphology and biochemistry, leading to the development of the storage lesion, which can confound studies on in vivo aging if stored blood is used for analysis. Obeidi et al. reported significant changes in red cell indices when testing was performed 24 h post sample collection [94].

## 9. Conclusions and Future Directions

RBCs undergo a multitude of morphological, functional, and biochemical changes with age, indicating their potential as biomarkers of human aging. Parameters such as RDW and MCV show promise as indicators of both chronological and biological age, while alterations in membrane proteins and metabolic markers further underscore the age-related changes occurring in these cells. The potential clinical applications of RBC characteristics in predicting age-related diseases and assessing health are significant, offering a readily accessible source of information about an individual’s aging trajectory.

While the accessibility and cost-effectiveness of RBC indices such as RDW is well acknowledged, their true value lies in how they can complement existing biological aging markers. Indeed, elevated RDW has been shown to predict all-cause mortality, cognitive impairment, and physical frailty, suggesting that it could act as an early warning marker before declines are evident in performance-based assessments.

Compared to molecular biomarkers such as epigenetic clocks, RDW offers a more dynamic and systems-level snapshot of the aging process, particularly in relation to oxygen transport, oxidative stress, and inflammation. While epigenetic clocks provide insight into the molecular underpinnings of biological aging, their high technical and financial barriers limit their scalability, particularly in resource-limited settings. The integration of RBC indices with molecular and functional assessments may enhance predictive accuracy by capturing both the molecular and systemic dimensions of aging, ultimately supporting more personalized and context-sensitive aging assessments.

However, several limitations and challenges must be carefully considered when utilizing RBCs as reliable biomarkers of aging. These include the limited functional repertoire of mature RBCs, inter-individual variability, the overlap between aging and disease processes, technical challenges in isolating age-specific RBC populations, the confounding effects of blood storage, and inconsistencies in the correlation of some RBC parameters with age.

Future research should focus on further elucidating the underlying molecular mechanisms that drive age-related changes in RBCs, potentially linking them to the broader hallmarks of aging. The development of more sensitive and specific RBC-based biomarkers for biological age and age-related diseases, possibly through the integration of multi-omics approaches and advanced analytical techniques, holds great promise. Longitudinal studies that track RBC parameters in individuals over extended periods are needed to better understand the dynamics of these age-related changes and their predictive power for health outcomes. Standardization of methods for RBC isolation, storage, and analysis is crucial to minimize confounding factors and improve the reliability and comparability of RBC biomarkers across different studies. Finally, exploring the potential for therapeutic interventions that target the aging processes within RBCs could pave the way for novel strategies to promote healthy aging and prevent age-related diseases.

Despite the existing limitations, RBCs remain valuable and accessible cellular components that offer a unique window into the aging process. Continued rigorous scientific investigation, with careful consideration of the inherent challenges, will be essential to fully realize the potential of RBCs as biomarkers in the field of aging research and to translate these findings into clinically meaningful applications for improving human health.

## Figures and Tables

**Table 1:** Comparison of erythropoiesis in young vs. aged humans.

Feature	Young Individuals	Aged Individuals
HSC function	High self-renewal and proliferation capacity	Reduced self-renewal; stem cell exhaustion
EPO	High responsiveness to EPO	Diminished EPO receptor signaling and responsiveness
Bone marrow microenvironment	Supportive niche with low inflammation	Increased adiposity, chronic low-grade inflammation
Iron availability	Adequate absorption and utilization	Functional iron deficiency due to hepcidin elevation
Inflammatory cytokines (e.g., IL-1, TNF-α, IFN-γ)	Low baseline levels	Elevated levels; suppression of erythroid progenitors
Erythropoiesis efficiency	Robust production and differentiation	Impaired differentiation and increased risk of anemia

**Table 2:** Morphological and functional changes in RBCs during organismal aging.

RBC Feature	Young Adults	Older Adults
RBC count	Normal/high	Decreased
Hb	Normal	Decreased
MCV	Normal	Slightly increased
RDW	Low (homogenous size)	Increased (heterogeneous size)
Deformability	High (good microcirculation)	Decreased (rigid, impaired flow)
ATP release	Adequate response to hypoxia	Reduced ATP-mediated vasodilation
Aggregation tendency	Low	Increased aggregation and blood viscosity
Oxygen carrying capacity	Efficient Hb saturation and delivery	Reduced capacity and altered Hb affinity
Membrane integrity	High fluidity, stable proteins	Increased lipid peroxidation, protein clustering
Antioxidant enzyme activity	High (protection against ROS)	Decreased, higher oxidative stress susceptibility

**Table 3:** Comparison of key biomarkers associated with aging.

Biomarker	Primary Mechanism Associated with Aging	Key Advantages	Key Limitations
Telomere Length	Cellular senescence and replicative history	Dynamic indicator of biological age Telomere length can be measured repeatedly and accurately	Evidence suggesting telomere length is a biomarker of aging in humans is equivocal High inter-individual variability Relatively complex/costly measurement
DNA Methylation (Epigenetic Clocks)	Epigenetic drift and gene regulation	Epigenetic changes can predict the aging process	The association between epigenetic modifications and age is not fully understood DNA methylation is highly cell-type specific Generalisability and specificity of epigenetic clocks across diverse populations pose a challenge
RBC indices	RBC integrity and function changes with age.	Accessible and cost-effective Reflects changes in multiple RBC parameters that correlate with aging RDW is a robust predictor of all-cause mortality	Less direct mechanistic link to primary aging processes

## Data Availability

Not applicable.
